# Image-guided fine-needle aspiration cytology of ovarian tumors: An assessment of diagnostic efficacy

**DOI:** 10.4103/0970-9371.71872

**Published:** 2010-07

**Authors:** Ghazala Mehdi, Veena Maheshwari, Sheerin Afzal, Hena A Ansari, Maryem Ansari

**Affiliations:** Department of Pathology, Jawaharlal Nehru Medical College and Hospital, Aligarh Muslim University, Aligarh, UP, India

**Keywords:** Image-guided FNAC, ovarian tumors, histopathology, diagnostic accuracy

## Abstract

**Background:**

Image-guided fine-needle aspiration cytology (FNAC) of ovarian lumps is being increasingly used for the successful diagnosis of ovarian tumors, although borderline cases may be difficult to diagnose by this method.

**Aim:**

To demonstrate the efficacy of image-guided FNAC in diagnosing ovarian tumors (benign and malignant) and to evaluate the usefulness of cytology as a mode of easy and rapid diagnosis of ovarian lumps.

**Materials and Methods:**

The study was conducted on 42 female patients. Clinical evaluation and relevant investigations were carried out. Diagnosis was established by FNAC performed under image guidance (ultrasonography/computed tomography). The cytological diagnosis was confirmed by histopathological examination.

**Results:**

Cytological diagnosis was rendered on all the 42 ovarian lesions, with a correct diagnosis in 34 cases, resulting in a diagnostic accuracy of 80.9%. Most of the cases with discordant diagnoses were surface epithelial tumors of low malignant potential and required histopathological examination for a final diagnosis.

**Conclusions:**

Image-guided FNAC is an inexpensive, rapid and fairly accurate procedure for the diagnosis of ovarian lesions. It provides a safe alternative to the more expensive, time consuming and cumbersome surgical route to diagnosis.

## Introduction

The clinicopathological evaluation of ovarian masses is a challenging field. Difficulty in gaining access to the tumor site is itself a major obstacle and the wide spectrum of lesions presents a daunting picture to the pathologist. Although histopathology remains the gold standard, in recent times, image-guided aspiration is being increasingly used as a rapid, inexpensive and efficient method for the pre-surgical diagnosis of ovarian masses as well as for planning and evaluation of treatment.

Aspiration under image guidance can be done with the use of ultrasonography (USG), computed tomography (CT) or magnetic resonance imaging (MRI). USG is a rapid, inexpensive and versatile technique. No ionizing radiation or injection of contrast medium is required and it can be repeated easily if required.[1] CT and MRI are costlier alternatives but are superior to USG in assessment of the nature of ovarian masses, the accuracy being highest with MRI.[2] It has been shown that these modalities have similar accuracy in assessment of the stage of disease,[3] although USG lags behind in the depiction of peritoneal metastases.[4] CT is better for diagnosing, staging and planning surgery in women with suspected metastatic spread of ovarian cancer.[4] However, CT is associated with a significant risk of radiation exposure.

FNAC under USG or CT guidance can be regarded as the investigation of choice for diagnosis of abdominal masses in the early stages of disease.[5] It can help in typing of uterine adnexal tumors, avoiding unnecessary surgical intervention.[6] The concurrent use of both the techniques (USG and CT) ensures greater accuracy in the assessment of tumors. Despite the obvious advantages, the frequent use of image-guided FNAC for routine investigation and diagnosis of ovarian neoplasms is a controversial field and has been the subject of much debate.

This study was undertaken to assess the diagnostic accuracy of image-guided (abdominal USG and CT) FNAC in the diagnosis of ovarian masses, as well as to assess the efficacy of cytology as a rapid and cost-effective means of diagnosing ovarian tumors.

## Materials and Methods

The study was conducted on 42 female patients who presented in the outpatient section of the Department of Obstetrics and Gynaecology and were subsequently found to have an ovarian mass on clinical and radiological evaluation. After clinical workup, the patients were subjected to abdominal/pelvic USG-or CT-guided FNAC. The mass was localized and aspiration performed using a 22-to 23-gauge needle attached to a 10 mL syringe. For deep-seated lesions, a lumbar puncture needle was used. Several passes were made when the needle was visualized within the lesion. Smears were prepared from the aspirate, fixed in 95% alcohol and stained using Papanicolaou and hematoxylin and eosin stains. Fluid aspirates were processed using the cytospin technique. No major complications were observed in any of the patients.

## Results

The majority of patients with ovarian masses presented in the second to fourth decade of life, with a peak in the fourth decade. The most common presenting features were an abdominal mass (90.5%) and lower abdominal pain (71.4%). Dyspareunia (11%) and menstrual disturbances (10%) were less common symptoms.

**Figure 1:**
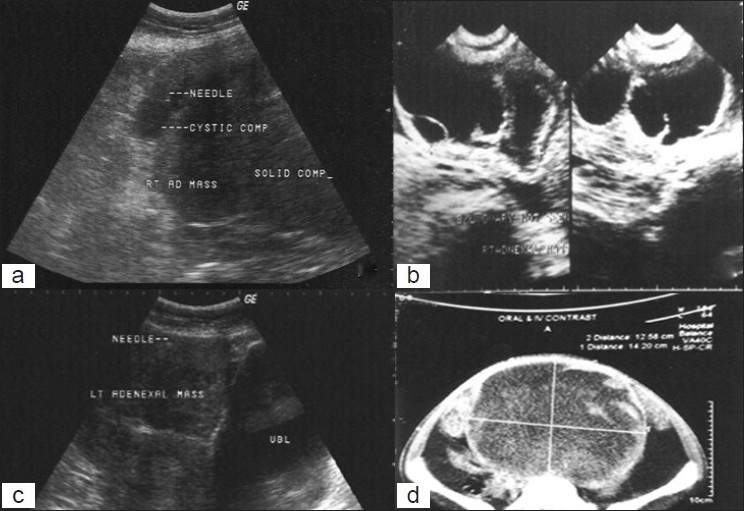
Ultrasonography scan showing (a) right adnexal mass (serous cystadenoma) with solid and cystic components (b) solid cystic mass involving both ovaries (bilateral serous cystadenocarcinoma) and (c) left adnexal mass with needle inside the lesion (d) contrast-enhanced computed tomography: large ovarian mass of mixed fat and soft tissue attenuation (teratoma)

USG/CT enabled an assessment of the type of lesion (whether solid or cystic), size, location and extent of the lesion [Figure 1a–d] and thus augmented the cytological diagnosis of the case.

Out of a total of 42 cases, the majority of cases were of surface epithelial tumors (35 cases). Twenty-five (59.5%) were diagnosed on cytology as benign. These included 14 cases (33.3%) of serous cystadenoma, 7 cases (16.7%) of mucinous cystadenoma and 4 cases (9.5%) of teratoma. The remaining 17 cases (40.5%) were diagnosed as malignant lesions and consisted of 10 cases (23.8%) of serous adenocarcinomas, 4 cases (9.5%) of mucinous adenocarcinomas and 3 cases (7.2%) of dysgerminoma. On cyto-histological correlation, it was observed that out of a total of 42 cases, 34 cases (80.9%) were accurately identified and sub-typed (19 benign and 15 malignant tumors) [Table 1].

**Table 1:** Cyto-histological correlation of ovarian tumors

Cytological diagnosis	Total (% of cases)	Histopathological diagnosis	
		Concordant (diagnostic accuracy)	Discordant
Benign	25 (59.5)	19 (76%)	6
Serous cystadenoma	14	11	Serous cystadenocarcinoma of low malignant potential (3)
Mucinous cystadenoma	7	5	Mucinous cystadenocarcinoma of low malignant potential (2)
Teratoma	4	3	Immature teratoma (1)
Malignant	17 (40.5)	15 (88.2%)	2
Serous adenocarcinoma	10	9	Mucinous adenocarcinoma (1)
Mucinous adenocarcinoma	4	3	Mucinous cystadenocarcinoma of low malignant potential (1)
Dysgerminoma	3	3	0
Total	42	34 (80.9%)	8 (19.1%)

**Figure 2:**
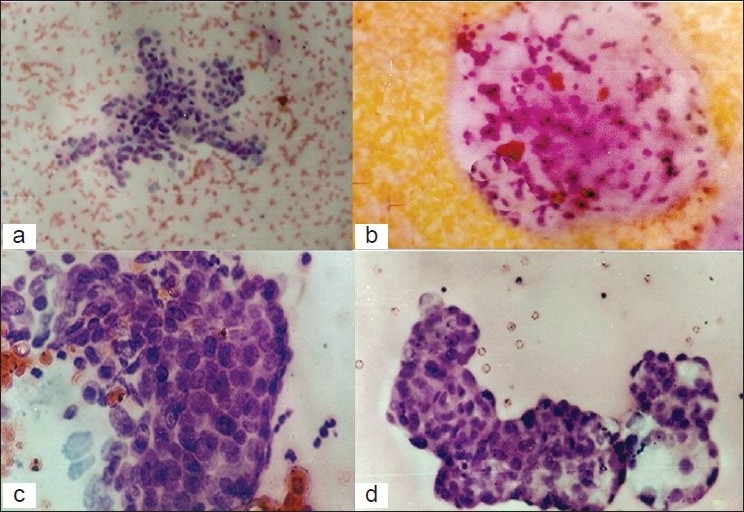
Fine-needle aspiration cytology (FNAC): (a) serous cystadenoma—papillary arrangement of benign epithelial cells (H and E, ×250) (b) mucinous cystadenoma—mucinous material with scattered columnar and inflammatory cells (H and E, ×250) (c) serous cystadenocarcinoma—papillary arrangement of malignant epithelial cells with large nuclei and prominent nucleoli (H and E, ×250) and (d) mucinous cystadenocarcinoma—tumor cells in a papillary pattern, with vacuolated cytoplasm and mucin secretion (H and E, ×250)

Straw colored fluid was aspirated in cases of serous cystadenomas. The FNAC smears were scantily cellular. A few papillary aggregates of the epithelial cells showing bland nuclei were seen in a background of foamy histiocytes and scattered inflammatory cells [Figure 2a]. In cases of mucinous cystadenomas, tall columnar cells with basally placed nuclei and vacuolated cytoplasm were observed against a mucinous background [Figure 2b]. Three cases of serous and 2 cases of mucinous cystadenocarcinomas of low malignant potential could not be identified correctly on cytological examination due to degenerative changes in cell morphology, resulting in a diagnostic accuracy of 78.5% and 71.5% for benign serous and mucinous tumors, respectively. Overall diagnostic accuracy was 76%.

The smears of malignant papillary serous cystadenocarcinomas showed good cellularity with papillary aggregates of tumor cells having large hyperchromatic nuclei and high nuclear–cytoplasmic (N/C) ratio, against a background of hemorrhagic fluid [Figure 2c]. Cytoplasmic vacuoles were noted in some cells. Occasional psammoma bodies were also seen. Diagnostic accuracy was 90% in this group. Cohesive sheets of columnar mucin-producing cells with nuclear enlargement, overcrowding, and a high N/C ratio (against a background of stringy mucin) were highly suggestive of mucinous cystadenocarcinoma [Figure 2d], yielding a diagnostic accuracy of 75%.

A single case of mucinous adenocarcinoma was diagnosed on cytology as a serous cystadenocarcinoma. This was probably because representative area of the tumor was not aspirated. Another case of mucinous adenocarcinoma of low malignant potential was also overdiagnosed as an invasive mucinous cystadenocarcinoma.

**Figure 3:**
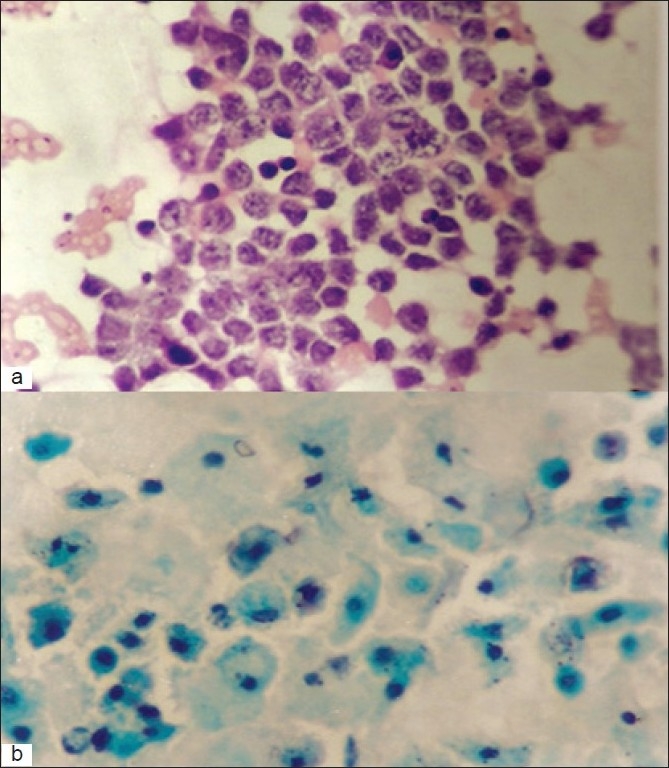
(a) Aspirate from a dysgerminoma showing loosely cohesive cells with scant cytoplasm, vesicular nuclei and prominent nucleoli. Scattered lymphocytes are also visible (H and E, ×250) (b) nucleate and anucleated squamous cells are seen in material aspirated from a teratoma, with amorphous material in the background (Pap, ×500)

In addition to surface epithelial tumors, we also evaluated 3 cases of dysgerminoma in our study, with 100% diagnostic accuracy on cytological smears. Cytologically, aspirates from dysgerminomas were characterized by the presence of dispersed tumor cells with pale, scant cytoplasm, large vesicular nuclei and accompanying lymphocytes [Figure 3a]. Four cases of teratoma yielded a greasy aspirate wherein nucleate and anucleate squamous cells along with inflammatory cells were seen [Figure 3b]. In one case, the final histopathological diagnosis was that of an immature teratoma.

Overall diagnostic accuracy was higher in the malignant category (88.2%) as compared with the benign tumors (76%). A concordant cyto-histological correlation was observed in 80.9% of cases. A total of 8 cases (19.1%) could not be accurately classified on cytology. It was noted that borderline epithelial tumors (6 cases) were most frequently misdiagnosed and classified as either their benign or malignant counterparts [Table 1].

## Discussion

Patients with ovarian tumors, particularly those with malignant neoplasms, usually present with advanced disease. Most of our patients presented with the complaints of lump in the abdomen and abdominal pain.

Benign ovarian lesions were observed in younger patients with an average age of 38 years. The youngest patient was a 2-year-old female child who was diagnosed with teratoma of the ovary. Malignant lesions were more frequent in the fourth decade (average age: 55 years).

We observed excellent cellular morphology in most cases, which facilitated the diagnosis and was in accordance with the documented literature.[7,8]

Tumors of low malignant potential or borderline tumors were difficult to diagnose with accuracy on cytological examination and often could not be clearly distinguished from well-differentiated cystadenocarcinoma[9–12] or even cystadenomas as was seen in 6 of our cases. This category of ovarian neoplasms constitutes a grey zone and is subject to inter-observer variations. Histopathology is a pre-requisite for assessing the presence or absence of stromal invasion and for the sub-typing of a tumor with low malignant potential. A high index of suspicion and careful evaluation of nuclear features is therefore essential.

Serous adenocarcinoma (including carcinomas of low malignant potential) was the most commonly diagnosed malignant ovarian tumor. While we noted a high rate of diagnostic accuracy in this category (90%), it is important to emphasize that mucinous areas of a mucinous cystadenocarcinoma must be sampled in order to avoid a false diagnosis of a serous tumor, as occurred in one case in our study.

Mucinous adenocarcinoma formed the second largest group of malignant ovarian neoplasms. Three cases were correctly diagnosed on cytology as mucinous adenocarcinoma, whereas 3 others were histologically diagnosed as mucinous cystadenocarcinomas of low malignant potential. We achieved a high rate of accuracy (80.9%) in diagnosing ovarian tumors, both benign and malignant. High rates of diagnostic accuracy have been reported by other workers also.[12–15]

Aspiration under image guidance for the evaluation of ovarian masses is justified as it is a relatively quick, economic and patient-friendly procedure, with minimal morbidity. With these modalities, any structure visualized radiologically can usually be reached precisely and in any desired plane. Nevertheless, as with any technique, image-guided FNAC has its shortcomings; false-negative results are usually due to failure of the needle to enter the mass and failure to sample representative areas.

The use of aspiration cytology to diagnose ovarian lesions has been widely discussed.[9–11,16] It is extremely useful in young patients with benign lesions, such as benign cysts, where an early diagnosis can help in avoiding surgery in some cases.[10,11,17] It also helps in minimizing unnecessary surgery in post-menopausal patients and those at high risk for surgery.[11]

In cases of malignant tumors, FNAC has a definitive role in evaluating patients with suspected recurrence of the tumor and to assess spread of the disease.[18,19] However, its use as a first-line diagnostic modality is debatable. Although there is no doubt about the accuracy of diagnosis, the major drawback is that FNAC can lead to rupture and spillage of tumor cells into the peritoneal cavity and can potentially cause upstaging of a malignant tumor.[20] It has been seen that USG can help in identifying tumors as benign or malignant.[21,22] This can serve as a useful guide in assessing which tumors should be aspirated and which should be taken up for surgical evaluation.

Despite the potential disadvantages, image-guided FNAC has an important role to play in the diagnosis and management of most ovarian masses. When combined with radiological assessment of the nature of the tumor, FNAC can serve as a highly efficient means of early diagnosis of ovarian neoplasms.

## References

[1] Porter B, Karp W, Forsberg L (1981). Percutaneous cytodiagnosis of abdominal masses by USG guided FNAB. Acta Radiol.

[2] Kurtz AB, Tsimikas JV, Tempany CMC, Hamper UM, Arger PH, Bree RL (1999). Diagnosis and staging of ovarian cancer: comparative values of Doppler and conventional US, CT, and MR imaging correlated with surgery and histopathologic analysis - report of the radiology diagnostic oncology group. Radiology.

[3] Tempany CMC, Zou KH, Silverman SG, Brown DL, Kurtz AB, McNell BT (2000). Staging of advanced ovarian cancer: comparison of imaging modalities-report from the radiological diagnostic oncology group. Radiology.

[4] Spencer JA (2005). A multidisciplinary approach to ovarian cancer at diagnosis. Br J Radiol.

[5] Ahmad SS, Akhtar K, Akhtar S, Abrari A, Nasir A, Khalid M (2006). Ultrasound guided fine needle aspiration biopsy of abdominal masses. JK Science.

[6] Ferran A, Gallardo J, Padilla C, Combalia N, Mellado F, Rey M (1995). Fine needle aspiration of benign uterine adnexal lesions. Acta Cytol.

[7] Ramzy I, Delaney M, and Rose P (1979). Fine needle aspiration of ovarian masses. II Correlative cytologic and histologic study of neoplastic cysts and non-coelomic epithelial neoplasms. Acta Cytol.

[8] Orell SR, Sterrett GF, Walters MNI, Whitaker D (2005). Fine needle aspiration cytology.

[9] Ganjei P (1995). fine needle aspiration cytology of the ovary. Clin Lab Med.

[10] Ganjei P, Dickinson B, Harrison TA, Nassiri M, Lu Y (1996). Aspiration cytology of neoplastic and non neoplastic ovarian cysts. Is it accurate?. Int J Gynecol Pathol.

[11] Wojcik EM, Selvaggi SM (1994). Fine needle aspiration cytology of cystic ovarian lesions. Diagn Cytopathol.

[12] Khan N, Afroz N, Aqil B, Khan T, Ahmad I (2009). Neoplastic and nonneoplastic ovarian masses: Diagnosis on cytology. J Cytol.

[13] Sood T, Handa U, Mohan H, Goel P (2010). Evaluation of aspiration cytology of ovarian masses with histopathological correlation. Cytopathology.

[14] Hemalatha AL, Divya P, Mamatha R (2005). Image-directed percutaneous FNAC of ovarian neoplasms. Indian J Pathol Microbiol.

[15] Kedar RP, Patel VH, Merchant SA, Aggarwal V, Pandit AA (1991). Ultrasound guided aspiration cytology-a valuable diagnostic aid. J Postgrad Med.

[16] Trimbos JB, Hacker NF (1993). The case against aspirating ovarian cysts. Cancer.

[17] De Crespigny LC, Robinson HP, Davoren RA, Fortune D (1989). The ‘simple’ ovarian cyst: Aspirate or operate?. Br J Obstet Gynaecol.

[18] Wojcik EM, Selvaggi SM (1992). Diagnostic accuracy of fine needle aspiration cytology in persistent or recurrent gynecologic malignancies. Diagn Cytopathol.

[19] Wojcik EM, Selvaggi SM, Johnson SC, Martier SS, Ager JW (1992). Factors influencing fine-needle aspiration cytology in the management of recurrent gynecologic malignancies. Gynecol Oncol.

[20] Bergman CA, Ozols RF (2003). Diagnosis and staging. Ovarian cancer., Atlas of clinical oncology (American cancer society).

[21] Sassone AM, Timor-Tritsch IE, Artner A, Westhoff C, Warren WB (1991). Transvaginal sonographic characterization of ovarian disease: Evaluation of a new scoring system to predict ovarian malignancy. Obstet Gynecol.

[22] Yee H, Greenebaum E, Lerner J, Heller D, Timor-Tritsch IE (1994). Transvaginal sonographic characterization combined with cytologic evaluation in the diagnosis of ovarian and adnexal cysts. Diagn Cytopathol.

